# Redescription of *Brochopeltis mjoebergi* Verhoeff, 1924 and description of a second *Brochopeltis* species from Australia (Diplopoda, Polydesmida, Paradoxosomatidae)

**DOI:** 10.3897/zookeys.504.9811

**Published:** 2015-05-18

**Authors:** Robert Mesibov

**Affiliations:** 1 Queen Victoria Museum and Art Gallery, 2 Invermay Road, Launceston, Tasmania 7248, Australia

**Keywords:** Diplopoda, Polydesmida, Paradoxosomatidae, Queensland, Northern Territory, Australia

## Abstract

*Brochopeltis mjoebergi* Verhoeff, 1924 is redescribed from type and new material, a lectotype is designated and *Brochopeltis mjoebergi queenslandica* Verhoeff, 1924 is synonymised with *Brochopeltis mjoebergi*. *Brochopeltis mediolocus* **sp. n.** is the first native paradoxosomatid described from Australia’s Northern Territory.

## Introduction

Paradoxosomatid species in the Australian genera *Brochopeltis* Verhoeff, 1924, *Helicopodosoma* Verhoeff, 1924 and *Tholerosoma* Mesibov, 2006 have unbranched gonopod telopodites with the prostatic groove opening at the telopodite tip. The taxonomic placement of these genera is uncertain. *Brochopeltis* and *Helicopodosoma* species have a medial process on the male leg 1 femur, a diagnostic feature of the subfamily Australiosomatinae. Both genera were placed in the tribe Antichiropodini within Australiosomatinae by Jeekel (1968). However, Jeekel (1979: 652) later suggested that *Brochopeltis*, *Helicopodosoma*, *Australodesmus* Chamberlin, 1920 and *Mjoebergodesmus* Verhoeff, 1924 were distinctive enough to merit each being placed in its own tribe. *Tholerosoma* species lack a process on the male leg 1 femur (Mesibov 2006), and I left the genus without a subfamily assignment in my original description.

The three genera are hard to place because the gonopod is so simple in structure. There are no obvious clues to intergeneric relationships in the non-gonopodal characters, and gonopod simplification could have occurred in more than one ancestral lineage. Verhoeff (1924: 33) clearly recognised this problem when discussing *Brochopeltis*: “...it is wrong to judge genera solely according to the gonopods, particularly when these, as is here the case, are so simplified through secondary regression of the tibiotarsus, that very similar organs could also arise in this way in related genera independently of one another” (my translation).

Gonopod simplification also makes it difficult to place a recently discovered species from Australia’s Northern Territory in a genus. In this paper I tentatively assign this species to *Brochopeltis* because of similarities to *Brochopeltis mjoebergi* Verhoeff, 1924 in both gonopod and paranota structure. I also redescribe *Brochopeltis mjoebergi* and designate a lectotype for it.

## Materials and methods

“Male” and “female” in the text refer to adult individuals. Specimens are stored in 70–80% ethanol in their respective repositories. Gonopods were cleared in 80% lactic acid and temporarily mounted in a 1:1 glycerol:water mixture for optical microscopy. Body measurements were estimated with a Nikon SMZ800 binocular dissecting microscope using an eyepiece scale. Colour images were manually stacked using a Canon EOS 1000D digital SLR camera mounted on the Nikon SMZ800 fitted with a beam splitter, then processed with Zerene Stacker 1.04. Figs 5B and 5C were captured as screenshots from the output of a 1.3 megapixel digital video eyepiece camera mounted in one ocular tube of a Tasco LMSMB binocular microscope. Preliminary gonopod drawings were traced from prints of screenshots captured in the same way. Images and drawings were prepared for publication using GIMP 2.8.

Suppl. material 1 tabulates data for known specimen lots of *Brochopeltis* species as of 15 April 2015 (data also available online in Mesibov 2006–2015). Locality details are given with latitude and longitude based on the WGS84 datum. My estimate of the uncertainty for a locality is the radius of a circle around the given position, in metres or kilometres. The locality map was generated using QGIS 1.75.

Abbreviations in text and Suppl. material 1: AM = Australian Museum, Sydney, Australia; ANIC = Australian National Insect Collection, Canberra, Australia; MAGNT = Museum and Art Gallery of the Northern Territory, Darwin, Australia; NHRS = Naturhistoriska Riksmuseet, Stockholm, Sweden; NT = Northern Territory, Australia; NTEIRC = Northern Territory Economic Insect Reference Collection, Darwin, Australia; Qld = Queensland, Australia; QM = Queensland Museum, Brisbane, Australia; ZMB = Museum für Naturkunde, Berlin, Germany; ZSM = Zoologische Staatssammlung München, Munich, Germany.

## Results

### Order Polydesmida Pocock, 1887 Suborder Strongylosomatidea Brölemann, 1916 Family Paradoxosomatidae Daday, 1889 Subfamily Australiosomatinae Brölemann, 1916 Tribe Antichiropodini Brölemann, 1916

#### Genus Brochopeltis

Taxon classificationAnimaliaPolydesmidaParadoxosomatidae

Verhoeff, 1924

Brochopeltis : Verhoeff 1924: 32; 1932: 1577, 1605. Attems 1926: 144; 1929: 261, 266; 1931: 137; 1937: 31, 275. Jeekel 1968: 20, 27, 30, 126; 1971: 218; 1979: 652, 654. Hoffman 1980: 166. Humphreys and Shear 1993: 181. Nguyen and Sierwald 2013: 1155.

##### Type species.

*Brochopeltis mjoebergi* Verhoeff, 1924, by monotypy.

##### Other assigned species.

*Brochopeltis mediolocus* sp. n.

#### Brochopeltis mjoebergi

Taxon classificationAnimaliaPolydesmidaParadoxosomatidae

Verhoeff, 1924

**Figure 1.**
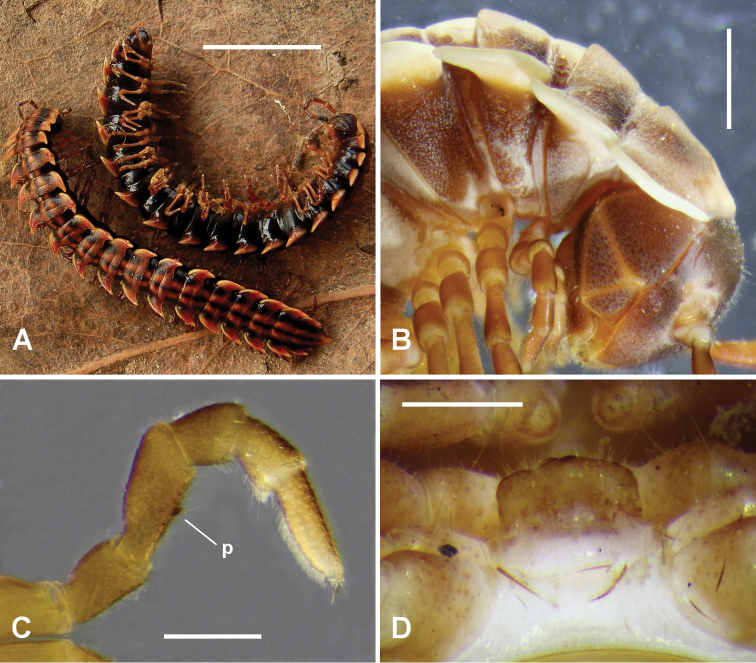
*Brochopeltis mjoebergi* Verhoeff, 1924. **A** female (top) and male (bottom) ex QM S74491 **B** male ex QM S74490. **A** habitus **B** right lateral view of anterior rings **C** anterior view of right leg 1 showing femoral process (**p**) **D** sternal lamella, posterior view. Scale bars: 10 mm (**A**); 1 mm (**B**); 0.5 mm (**C, D**).

**Figure 2.**
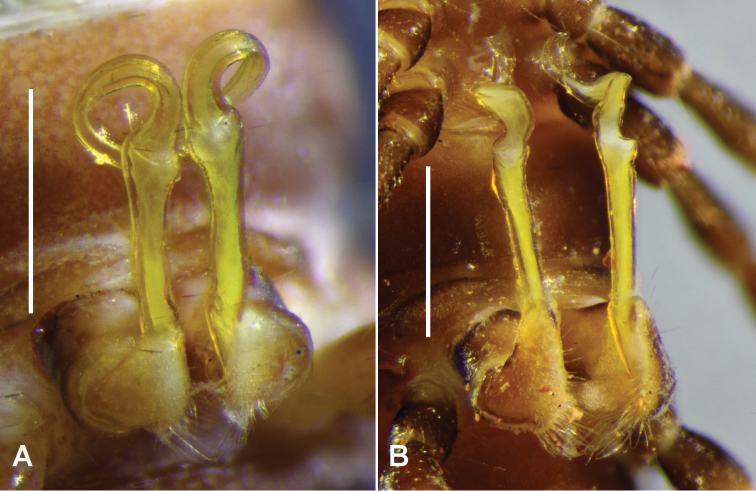
**A** *Brochopeltis mjoebergi* Verhoeff, 1924, male ex QM S74490 **B** *Brochopeltis mediolocus* sp. n., male paratype, NTEIRC 63895. Right ventrolateral views of gonopods in situ. Scale bars = 1.0 mm.

**Figure 3.**
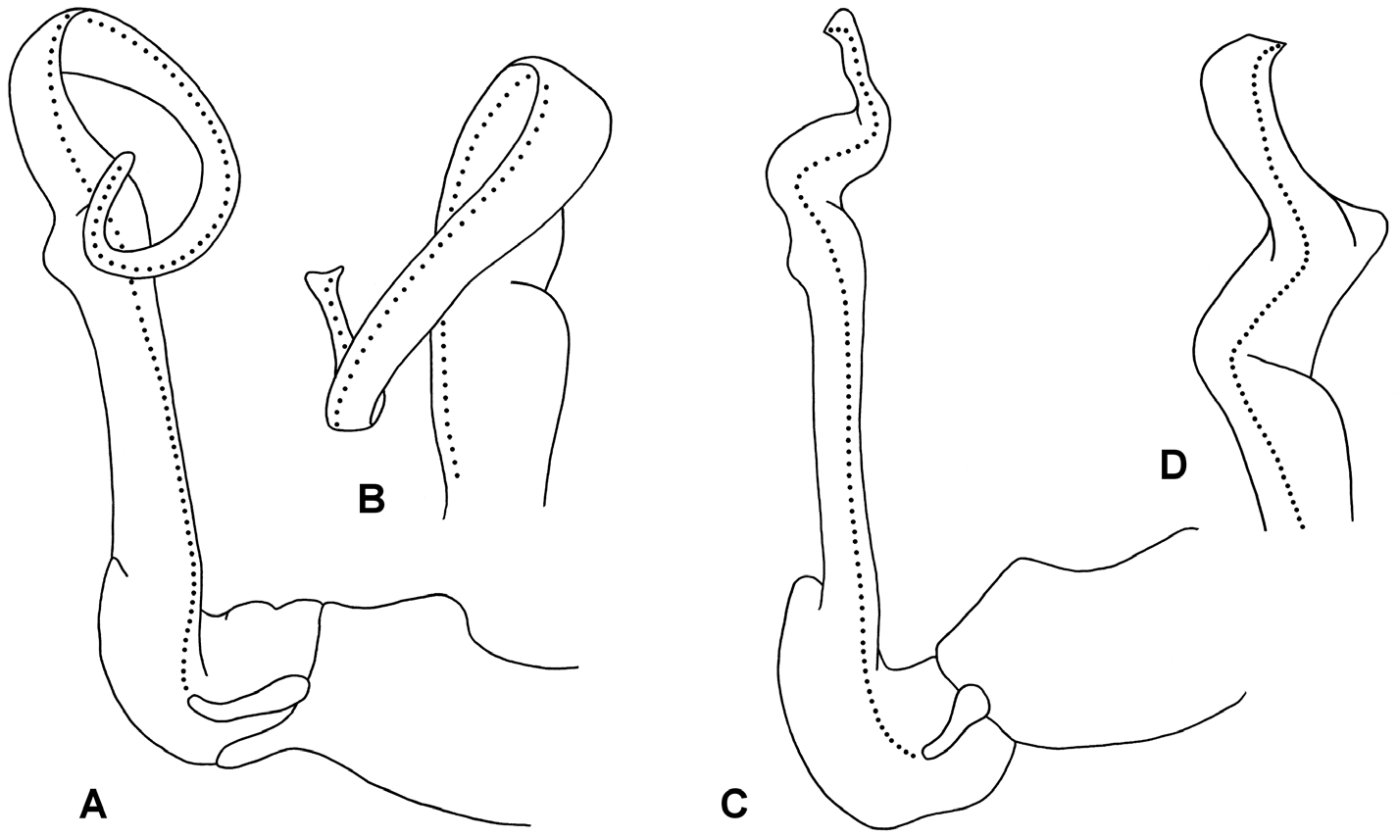
Right gonopods of *Brochopeltis mjoebergi* Verhoeff, 1924, male ex QMS 74490 (**A, B**), and *Brochopeltis mediolocus* sp. n., male ex NTEIRC 63897 (**C, D**) **A, C** Medial views **B** anterolateral view **D** anterior view. Drawings not to scale, setation not shown; dotted lines indicate course of prostatic groove.

**Figure 4.**
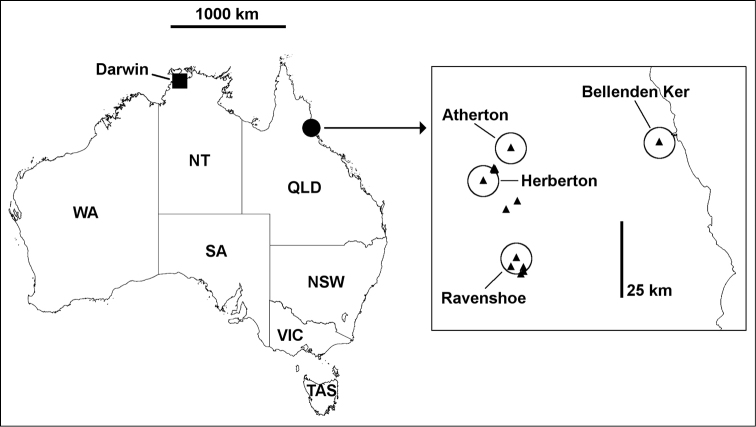
**Main map.** Locality for *Brochopeltis mediolocus* sp. n. (square) and locality area for *Brochopeltis mjoebergi* Verhoeff, 1924 (circle). **Inset.** Localities for *Brochopeltis mjoebergi* Verhoeff, 1924 (triangles) in far north Queensland; type localities are named and buffered with 5 km-radius circles. Mercator projections.

**Figure 5.**
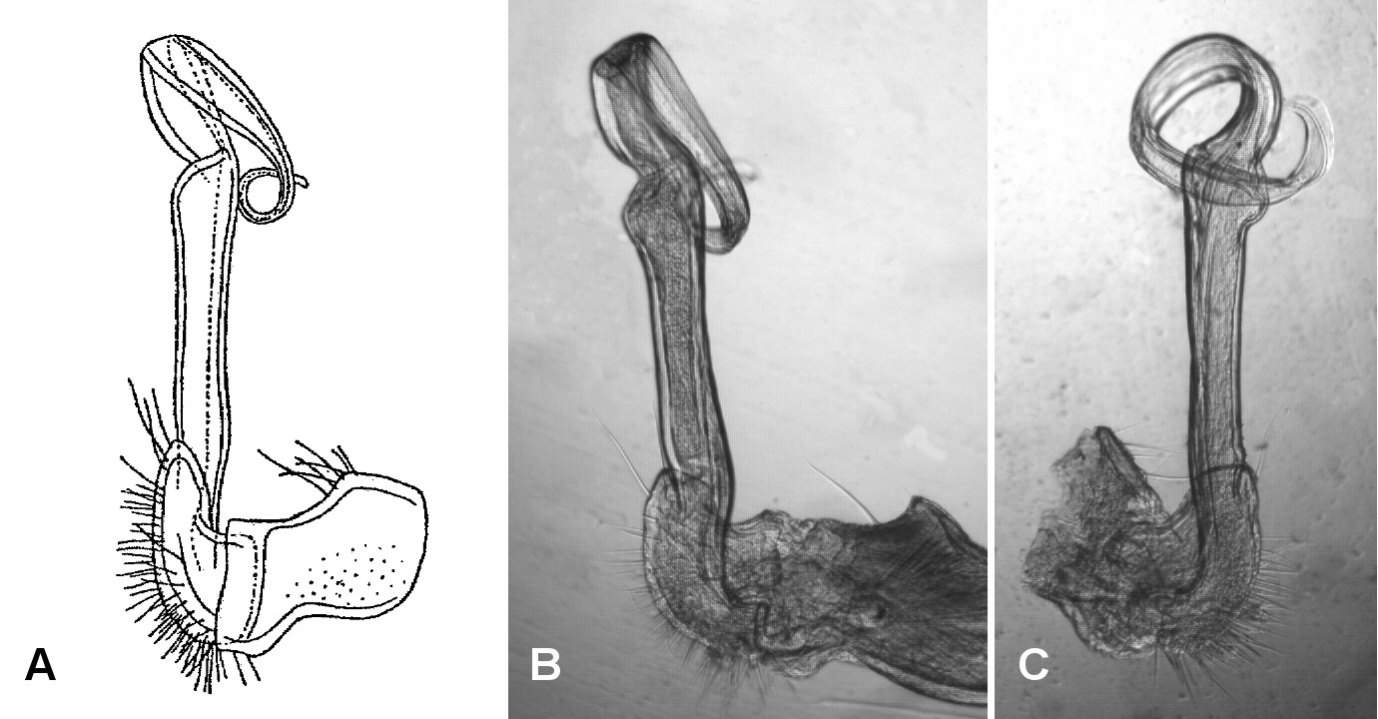
**A, B** *Brochopeltis mjoebergi* Verhoeff, 1924 **C** *Brochopeltis mjoebergi queenslandica* Verhoeff, 1924 **A** Lateral view of left gonopod of syntype, from Verhoeff (1924), fig. 20 **B** Medial view of right gonopod of paralectotype as mounted on slide by Verhoeff, NHRS slide 266, KASI000000026 **C** Lateral view of right gonopod of lectotype as mounted on slide by Verhoeff, NHRS slide 265, KASI000000029. Images not to same scale.

Figs 1 , 2A , 3A , 3B , 5 ; Fig. 4 (map)

Brochopeltis mjöbergi : Verhoeff 1924: 33 (misprinted here as *mjöbergii*; see Remarks), Fig. 20 in pl. 2; 1932: Fig. 980 (p. 1597). Attems 1937: 275, 276, Fig. 343 (p. 275).
Brochopeltis mjoebergi : Jeekel 1968: 19, 30; 1971: 218. Nguyen and Sierwald 2013: 1155.
Brochopeltis mjöbergi queenslandica : Verhoeff 1924: 35. Attems 1937: 276. **syn. n.**
Brochopeltis mjoebergi queenslandica : Jeekel 1968: 19, 30. Nguyen and Sierwald 2013: 1155. **syn. n.**

##### Lectotype

**(here designated).** 1 male, Herberton, Qld, E. Mjöberg, 1913, NHRS KASI000000031, in 2 pieces in separate vial.

##### Paralectotypes.

**NHRS:** 2 entire females and parts of 3 males, 2 females and 1 juvenile, males with gonopods intact, collecting details as for lectotype, KASI000000031, in alcohol with printed label “Queensl. / Mjöberg” and Verhoeff labels “Brochopeltis / mjöbergi Verh. / Herberton” (in pencil) and “Brochopeltis Mjöbergi Verh. / Queensl. Herberton. / [Colleg.] Mjöberg. [Determ.] Verhoeff.” (in pen); slide mount of 1 right and 1 left gonopod and 2 male eighth legs, Atherton, Qld, same collector and year, slide 266, KASI000000026, Verhoeff label “Brochopeltis mjöbergi Verh. / Atherton, 8.B. / Queensland. a2”, Johns label “Lectotype ♂ / parts of body in alcohol. / P.M. Johns 10.viii.67”; 1 male, body broken into 4 parts and missing ring 7, same collecting details, KASI000000026, in alcohol with Johns label “Lectotype ♂ / genitalia on slide / P.M. Johns 10.viii.67”; slide mount of 1 right and 1 left gonopod, same collecting details, NHRS slide 267, KASI000000028, Verhoeff label “Brochopeltis mjöbergi Verh. / Atherton. / Queensland. a1”, Johns label “Paralectotype ♂ / Body in alcohol / P.M. Johns 10.viii.67”; 2 entire females and parts of at least another 3 females, same collecting details, KASI000000028, in alcohol with printed labels “Queensl. / Mjöberg” and “Jan.”, label in pencil “Atherton / scrub / Jan 1913”, Verhoeff label in pen “Brochopeltis Mjöbergii Verh. / Atherton. Januar. /[Colleg.] Mjöberg. [Determ.] Verhoeff.”, Johns label “Paralectotypes 5♀♀ / P.M. Johns 10.viii.67”, also a smaller vial with rings 7-8 and 9? from 1 male, ring 7 without gonopods, Johns label “Paralectotype ♂ / genitalia on slide / P.M. Johns 10.viii.67”; parts of 3 females, same collecting details, KASI000000027, in alcohol with two printed labels “Queensl. / Mjöberg”, Verhoeff label “Brochopeltis / mjöbergi Verh. / Atherton” (in pencil) and Johns label “Paralectotypes 3♀♀ / P.M. Johns 10.viii.67”. **ZMB (not examined):** 1 male, Queensland, 1913, E. Mjöberg, ZMB 5710 (listed in Moritz and Fischer 1978; see Remarks). **ZSM (not examined):** 1 specimen in alcohol, Atherton, Qld, January 1913, E. Mjöberg, ZSM/Myr-20033548.00; 2 specimens in alcohol, same details, ZSM/Myr-20052193.00; 1 right and 1 left gonopod and 2 male first legs mounted on slide, same details, ZSM-A-20033548.

##### Lectotype of *Brochopeltis mjoebergi queenslandica*

**(here designated).** Male, Bellenden Ker, Qld, E. Mjöberg, 1913, comprising (1) slide mount of 1 right and 1 left gonopod, slide 265, KASI000000029, Verhoeff label “Brochopeltis / mjöbergi / queenslandica / Verh. / Bellenden Ker. / Queensland b1”, Johns label “Lectotype ♂ / body in alcohol / P.M. Johns 10.viii.67”, and (2) body in alcohol in small vial, broken between rings 5 and 6 and rings 9 and 10, KASI000000029, Johns label “Lectotype ♂ / genitalia on slide / P.M. Johns 10.viii.67”.

##### Paralectotype of *Brochopeltis mjoebergi queenslandica*.

**NHRS:** Male, collecting details as for lectotype, in alcohol in small vial, body broken into four parts, ring 7 isolated and with intact gonopods, KASI000000030, printed label “Queensl. / Mjöberg”, Johns label “Paralectotype / P.M. Johns 10.viii.67”.

*Brochopeltis mjoebergi queenslandica* lectotype and paralectotype vials in larger vial with two Verhoeff labels “Brochopeltis mjöbergi / queenslandica Verh. / Bellenden Ker” (in pencil) and “Brochopeltis Mjöbergi queens- / Bellenden Ker. landica Verh. / [Colleg.] Mjöberg. [Determ.] Verhoeff.” (in pen).

##### Other material.

25 males, 15 females and 1 juvenile in **AM**, **ANIC** and **QM** (see Suppl. material 1 for details).

##### Description.

(Based on lectotype and specimens collected 5-10 km from type locality in 1998.) Male/female approximate measurements: length 35/38 mm, midbody paranota width 5.2/5.4 mm, prozonite width 3.5/4.4 mm, maximum vertical diameter 3.5/4.4 mm. Well-coloured animals (Fig. 1A) very dark brown (almost black) on flanks and along narrow, longitudinal, mid-dorsal band, lighter reddish brown ventrally and in broad, paramedian, dorsal bands; paranota with pale margins; head and antennae dark brown, the antennae lighter basally; legs medium brown; body colour fades with long-term storage in alcohol.

Male with vertex and frons sparsely setose, clypeus moderately setose; vertigial sulcus distinct, ending at dorsal level of antennal sockets; post-antennal groove shallow; antennal sockets separated by ca 1.3× socket diameter. Antenna filiform, reaching dorsally to rear of ring 3; antennomeres with relative lengths (2=3)>(4=5=6); 6 apically widest. In dorsal view, head narrower than collum paranota; relative ring widths collum < (2=3=4) < (5 to17). Collum with lateral margin strongly produced as paranotum, anterior and lateral margins smoothly convex, posterior margin more or less straight. Paranota on haplo- and diplosegments with margins thickened dorsally, so dorsal paranotal surface appears slightly depressed. Ring 2 paranotum with lateral margin slightly lower than lateral margins of collum and ring 3 paranota (Fig. 1B); posterior corner produced posteriorly as broad triangle. Paranota of rings 3 and 4 curving posteriorly, almost sickle-shaped, posterior corners bluntly rounded. Paranota on diplosegments 5-17 set at ca 3/4 ring height, directed slightly dorsally with posterior corner highest; anterior margin curving smoothly into nearly longitudinal lateral margin, posterior corner strongly produced as bluntly pointed triangle almost reaching waist of next ring; lateral margin thicker on pore-bearing rings. Paranota reduced but still prominent on rings 18 and 19, with strongly produced posterior corners. Pleural keels on rings 2-4 only, on rings 3, 4 reduced to small, rounded, posterolaterally directed processes. Prozonites and metazonites bare and finely textured, giving dull appearance; transverse furrow at ca 1/2 metazonite length, distinct, ca 2/3 ring width, not reaching paranotal base; waist short, shallow, faintly sculptured with low longitudinal ridges; limbus a narrow, thin, continuous sheet. Pore formula normal; ozopore small, round, opening laterally on thickened paranotal margin almost at level of posterior metazonite margin. Spiracles on diplosegments above and just anterior to leg bases; spiracular filters forming rounded fold in inverted, tight U-shape in spiracular opening; anterior spiracle with anterodorsal portion of rim produced to partly cover strongly emergent, anterodorsal portion of filter. Midbody sternites very sparsely setose, as wide as long, transverse impression more distinct than longitudinal impression; no cones or projections on any sternites. Midbody legs slender, relative podomere lengths femur>tarsus>prefemur>(postfemur= tibia); femur ca 1.3× as long as tarsus; anterior leg prefemora not swollen dorsally. Pre-anal ring sparsely setose; epiproct extending past anal valves, in dorsal view tapering and truncate, tip ca 1/4 width of pre-anal ring; hypoproct paraboloid; spinnerets in rectangular array, much wider than long.

Leg 1 (Fig. 1C) with very small, rounded process at ca 2/3 length of medial femur surface, directed slightly anteromedially. Gonopore small, round, opening on short distomedial bulge of leg 2 coxa. Sternal lamella (Fig. 1D) ca 90% of width between leg 4 bases, short, more or less vertical; lateral margins straight, vertical; corners rounded; ventral margin very slightly convex. Dense brush setae on tarsi of legs 1-7 only.

Gonopod aperture just wide enough to accommodate gonocoxae, 1/3-1/2 ring 7 prozonite width. Gonopod telopodites (Figs 2A, 3A, B) parallel, almost reaching leg 6 bases when retracted; sternite between legs 6 and 7 bases slightly excavate. Rounded, transverse ridge just anterior to aperture on either side, the two ridges confluent medially.

Gonocoxa short, the anterodistal surface with low, rounded protuberance bearing sparse, long setae on distal side. Prefemur large, C-shaped, the distal end projecting posterolaterally as rounded extension reaching ca 1/5 telopodite height; long setae on posterior and posteromedial surfaces of prefemur. Cannula small, arising from gonocoxa apex. Telopodite beyond prefemur without branches, the basal portion straight and slightly expanded distally; at ca 2/3 telopodite height, telopodite constricted, flattening and curving anterolaterally in wide spiral to level of starting point of curve and anterior to it, then curving anterolaterally, the apex slightly expanded with distal margin rounded. Prostatic groove (Figs 3A, 3B) running straight on medial surface of basal portion of telopodite beyond prefemur, then following curve of telopodite to open at apex.

Female without leg modifications; epigynum 1/4-1/3 ring width, slightly raised in small rounded triangle medially; cyphopods not examined.

##### Distribution.

In forest litter within a range envelope of ca 1500 km^2^ on and near the Atherton Tableland, in the Wet Tropics of far north Queensland (Fig. 4).

##### Remarks.

*Types*. Verhoeff (1924: 35) reported that apart from a few pairs of *Brochopeltis mjoebergi* (“[a]usser einigen Pärchen”) from Atherton collected in January, he examined three males and four females from Herberton and one male and a juvenile from “Cedar Creek” (Ravenshoe). *Brochopeltis mjoebergi queenslandica* was based on two males from Bellenden Ker. All collections were by Erik Mjöberg in early 1913 (Ferrier 2006).

Most of the syntypes are accounted for, with a few discrepancies. The NHRS material comprises parts of two males and eight females from Atherton; parts of four (not three) males, four females and a juvenile from Herberton; and parts of two males from Bellenden Ker. ZSM has parts of at least two males from Atherton (J. Spelda, in litt.), and ZMB has one male labeled “Queensland” collected by Mjöberg in 1913 (J. Dunlop, in litt.). The latter may be the Ravenshoe male but if so the Ravenshoe juvenile appears to be missing.

The lectotypifications by P.M. Johns (see label information in types section, above) were never published. I have designated an entire male from Herberton in the type series as the *Brochopeltis mjoebergi* lectotype because the slide-mounted gonopods of Atherton males are distorted (see also below, on subspecies *queenslandica*).

*Species epithet*. The spelling *mjöbergii* on p. 33 in Verhoeff (1924) is apparently a typesetting error. The name is spelled *mjöbergi* on pp. 35, 133 and 138 with *Brochopeltis*, and an additional 26 times in Verhoeff (1924) with the new taxa *Cyliosoma queenslandicum mjöbergi*, *Monographis mjöbergi*, *Poratobolus mjöbergi*, *Rhinotus mjöbergi* and *Siphonophora mjöbergi*. The spelling *mjöbergi* is also used on all but one of the handwritten Verhoeff labels I have seen.

*Subspecies* queenslandica. Verhoeff distinguished this subspecies mainly on minor variation in colour pattern, writing “Structure otherwise as in the preceding form, the gonopods also agreeing with those of the other, but the solenomere bends not sharply bent, but totally rounded, thus even more strongly spirally curved” (Verhoeff 1924: 35, my translation). The supposed gonopod difference is an artefact produced during slide preparation (Fig. 5). Verhoeff mounted the spiral acropodites of the gonopods of the *mjoebergi queenslandica* lectotype more or less in the plane of the spiral, while those of the *mjoebergi mjoebergi* paralectotypes are mounted with the spirals compressed and bent. The intact gonopods of the *mjoebergi queenslandica* paralectotype spiral in just the same way as gonopods of *mjoebergi mjoebergi* from Herberton. The subspecies *queenslandica* is here made a synonym of the nominate subspecies.

*Other notes*. *Brochopeltis mjoebergi* appears to be abundant in rainforest and open forest on the western side of the Atherton Tableland (Fig. 4). I have not seen this large and easily recognised millipede in collections from elsewhere in the Queensland Wet Tropics. The region has been entomologically well sampled since Mjöberg’s expedition and is home to many other described and undescribed paradoxosomatids.

An anonymous Australian collector who calls *Brochopeltis mjoebergi* “fire millipede” posted a YouTube video in August 2014 documenting how this species can be kept in captivity (https://www.youtube.com/watch?v=hg6IlPF0YCo; accessed 9 March 2015).

#### Brochopeltis mediolocus

Taxon classificationAnimaliaPolydesmidaParadoxosomatidae

Mesibov sp. n.

http://zoobank.org/84765FF0-6D65-4687-80E9-B93028BE6059

**Figure 6.**
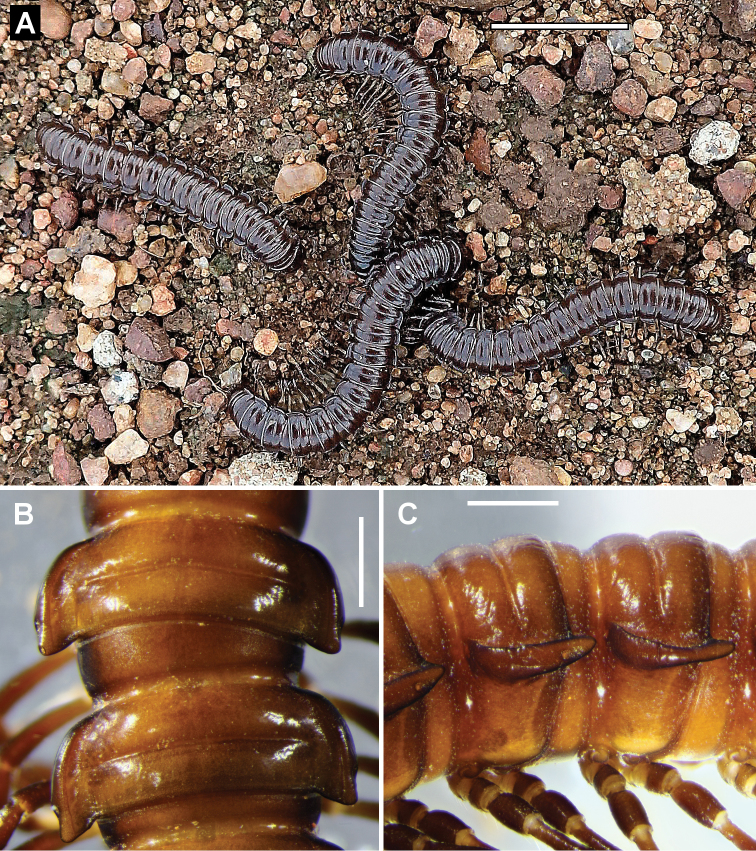
*Brochopeltis mediolocus* sp. n. **A** Live individuals at type locality, February 2015; image by Michael Neal **B** Dorsal and **C** lateral views of midbody rings of male paratype ex NTEIRC 63897. Scale bars: 10 mm (**A**)(approximate); 1 mm (**B, C**).

**Figure 7.**
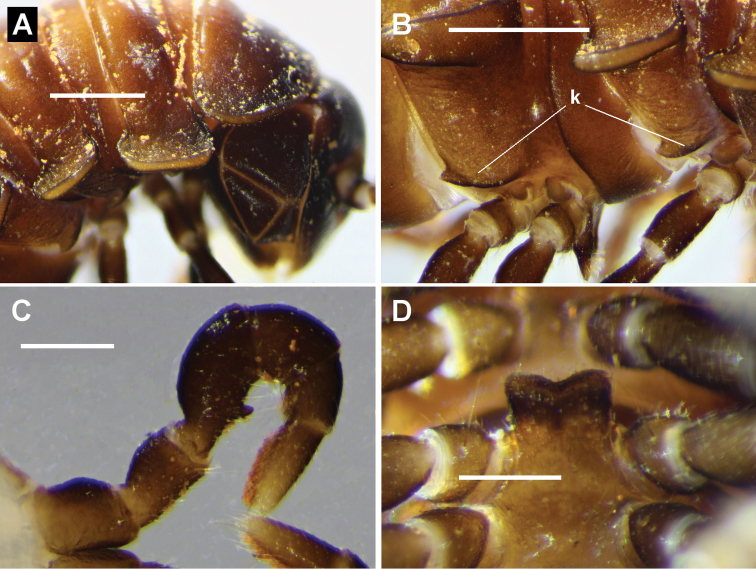
*Brochopeltis mediolocus* sp. n. **A–C** male paratype, NTEIRC 63895 **D** male paratype ex NTEIRC 63897 **A** Right lateral view of anterior rings **B** right lateral view of pleural keels (**k**) on rings 4 and 5, anterior to right **C** right leg 1, anterior view **D** sternal lamella, posteroventral view. Scale bars: 1 mm (**A, B**); 0.5 mm (**C, D**).

Figs 2B , 6 , 7 ; Fig. 4 (map)

##### Holotype.

Male, Anzac Parade (turf farm), Middle Point, NT, -12.5677 131.319 ± 200 m, 18 February 2015, M. Neal, ex ground at edge of pasture, MAGNT NTM-M000056.

##### Paratypes.

**NTEIRC:** 2 males, details as for holotype, 63897; 5 females, 13 juveniles, details as for holotype, 63898; 1 male, same details but 16 February 2015, in large numbers on ground, 63895.

##### Other material.

**NTEIRC:** 5 females, 117 juveniles (collected with types, not examined; see Suppl. material 1 for details).

##### Diagnosis.

Differs from *Brochopeltis mjoebergi* in distal portion of gonopod telopodite bent but directed distally, not curving in wide spiral; with dorsum uniform in colour, not with pale, paramedian longitudinal bands and pale paranota on a darker background; and with tarsal brushes on all male legs except last two pairs, rather than on legpairs 1-7 only.

##### Description.

Male/female approximate measurements: length 29/30 mm, midbody paranota width 3.6/3.8 mm, prozonite width 2.7/3.0 mm, maximum vertical diameter 2.6/3.0 mm. Live, well-coloured animals more or less uniformly dark brown in body colour, shiny (Fig. 6A). In alcohol, body colour brown (Fig. 6B, C), darker on paranota and posterior metazonite margin, lighter ventrally and in pleural keel area; head and antennae dark brown with lighter spot just above antennal socket; legs darker than body.

Male with vertex and frons almost bare, clypeus sparsely setose; vertigial sulcus distinct, ending at dorsal level of antennal sockets; post-antennal groove moderately deep; antennal sockets separated by ca 1.3× socket diameter. Antenna filiform, reaching dorsally to rear of ring 3; antennomeres with relative lengths (2=3)>(4=5)>6 and with 5 and 6 subequal in apical width. Head slightly narrower than collum in dorsal view, both narrower than ring 2; rings 2-17 subequal in width. Collum D-shaped in dorsal view, the lateral margin lifted slightly as a narrow paranotum, posterior corner rounded. Paranota on haplo- and diplosegments with margins thickened dorsally, so that dorsal paranotal surface appears slightly depressed. Ring 2 paranotum (Fig. 7A) with lateral margin lower than lateral margins of collum and ring 3 paranota; subtrapezoidal with rounded corners, extended slightly anteriorly and posteriorly. Ring 3 paranotum shorter than ring 2 paranotum; posterior corner extending posteriorly, rounded. Ring 4 paranotum intermediate in length between paranota of rings 2 and 3; posterior corner slightly extended posteriorly. Paranota on diplosegments 5-17 (Fig. 6B, C) set at ca 1/2 ring height; anterior corner strongly rounded; lateral margin further from the body posteriorly, thicker on pore-bearing rings; posterior corner rounded, progressively extending further posteriorly and passing posterior metazonite edge from about ring 10. Paranota greatly reduced but still prominent on rings 18, 19. Pleural keels (Fig. 7B) distinct on rings 2-8, reduced posteriorly to progressively smaller bulges, not detectable on rings 16-19; keels on rings 3-8 with well-defined lateral margins with posterior corners projecting a little posteriorly. Prozonites and metazonites (Fig. 6B, C) smooth, bare; transverse furrow at ca 1/2 metazonite length, distinct, extending laterally to paranotal base; waist short, shallow, not obviously sculptured; limbus a narrow, thin, continuous sheet. Pore formula normal; ozopore small, round, opening laterally on thickened paranotal margin almost at level of posterior metazonite margin. Spiracles on diplosegments above and just anterior to leg bases; anterior spiracle subquadrangular, posterior spiracle subtriangular; spiracular rim low, filter slightly emergent, forming rounded fold in inverted, tight U-shape in spiracular opening. Midbody sternites very sparsely setose, as wide as long, transverse impression wider than longitudinal impression; no cones or projections on any sternites. Midbody legs with relative podomere lengths femur>>(tibia=tarsus)>(prefemur=postfemur); femur ca 1.7× as long as tarsus; anterior leg prefemora only slightly swollen dorsally. Pre-anal ring sparsely setose; epiproct extending past anal valves, in dorsal view tapering and truncate, tip ca 1/5 width of pre-anal ring; hypoproct rounded-trapezoidal; spinnerets in rectangular array, wider than long.

Leg 1 (Fig. 7C) with small, short, rounded process at ca 1/2 length of medial femur surface, directed mediodistally and slightly anteriorly. Gonopore small, round, opening on short distomedial bulge of leg 2 coxa. Sternal lamella (Fig. 7D) ca 90% of width between leg 4 bases, leaning slightly anteriorly; lateral margins straight, vertical; corners rounded; ventral margin medially incised. Dense brush setae on tarsi of all but last 2 legpairs, on some anterior legs also at distal end of tibia.

Gonopod aperture just wide enough to accommodate gonocoxae, 1/3–1/2 ring 7 prozonite width. Gonopod telopodites (Figs 2B, 3C, D) straight, parallel, reaching leg 7 bases when retracted; sternite between leg 7 bases slightly excavate. Rounded, transverse ridge just anterior to aperture on either side, the two ridges nearly confluent medially.

Gonocoxa short, the anterodistal surface with low, ridge-like protuberance bearing sparse, long setae on distal side. Prefemur large, C-shaped, the distal end projecting posterolaterally as rounded extension reaching ca 1/4 telopodite height; numerous long setae on posterior and posteromedial surfaces of prefemur. Cannula small, arising from gonocoxa apex. Telopodite beyond prefemur without branches, the basal half straight and slightly expanded distally; at ca 2/3 telopodite height, telopodite flattening slightly and curving anterolaterally, then constricting and bending sharply anteriorly, curving mediodistally and flattening further, the apical margin rounded distally with lateral margin produced as small triangle. Prostatic groove (Fig. 3C, D) running straight on medial surface of basal half of telopodite beyond prefemur, then following bends and curves of telopodite to open at tip of apical triangular projection.

Female without leg modifications; epigynum ca 1/4 ring 2 width, very slightly raised medially in small rounded triangle; cyphopods not examined.

##### Distribution.

So far known only from the type locality, a farm ca 50 km southeast of Darwin in the monsoon tropics of Australia (Fig. 4).

##### Name.

Latin *medius*, “middle”, + *locus*, “place”, for the type locality, Middle Point; noun used as adjective.

##### Remarks.

I am tentatively assigning this species to *Brochopeltis* not only because the gonopods are similar, but because *Brochopeltis mediolocus* sp. n. and *Brochopeltis mjoebergi* share two features which I have not yet noted in other Australian Antichiropodini. One is the lifting and extension of the lateral collum margins as paranota. The second possible synapomorphy is the pronounced dorsal thickening of paranotal margins.

The types were collected on a farm and it is possible that *Brochopeltis mediolocus* sp. n. is not locally native, but has been introduced to Middle Point from elsewhere in tropical Australia. The only previous record of Paradoxosomatidae from the northern portion of the Northern Territory (Australia’s “Top End”) is of the introduced Asian species *Orthomorpha coarctata* (De Saussure, 1860) in urban Darwin (Jeekel 1982).

## Supplementary Material

XML Treatment for Brochopeltis

XML Treatment for Brochopeltis mjoebergi

XML Treatment for Brochopeltis mediolocus

## Supplementary materials

Supplementary material 1 Specimen records Data type: Tab Separated Value File (TSV) Explanation note: Specimen records of *Brochopeltis* species as of 15 April 2015. This dataset is made available under the Open Database License (http://opendatacommons.org/licenses/odbl/1.0/). The Open Database License (ODbL) is a license agreement intended to allow users to freely share, modify, and use this Dataset while maintaining this same freedom for others, provided that the original source and author(s) are credited.Robert Mesibov
